# Breast Cancer Brain Metastases: A Neurosurgical Point of View From a Single-Center Experience

**DOI:** 10.7759/cureus.82306

**Published:** 2025-04-15

**Authors:** Ligia G Tataranu

**Affiliations:** 1 Neurosurgery, Carol Davila University of Medicine and Pharmacy, Bucharest, ROU; 2 Neurosurgery Department, Emergency Clinical Hospital "Bagdasar-Arseni", Bucharest, ROU

**Keywords:** brain metastases, breast cancer, multimodal approach, neurosurgery, outcomes

## Abstract

Background: Brain metastases represent an important factor in breast cancer morbidity and mortality. Although various therapeutic options improved these patients’ outcomes, the incidence of this disease is still rising. Several molecular subtypes of breast cancer have been studied, and human epidermal growth factor receptor 2 (HER2) positive and triple-negative breast cancer (TNBC) are more frequently associated with brain metastases. Therefore, anti-HER2 agents have been developed and studied, and they have shown promising results. Nevertheless, in patients with breast cancer brain metastases and acute neurological aggravation, neurosurgery is the primary option and the only one that can immediately reverse the symptoms. In the long run, a multimodal approach involving neurosurgical intervention can positively impact the prognosis.

Material and methods: Patients with a confirmed diagnosis of brain metastases from breast cancer (BMBC) between January 2013 and December 2023 were retrospectively reviewed. All patients were newly diagnosed and treated in the 3rd Neurosurgical Department at the Clinical Emergency Hospital, “Bagdasar-Arseni” in Bucharest, Romania. Statistical analyses were carried out and interpreted accordingly.

Results: The study analyzed 62 patients with BMBC. The median age at diagnosis was 57.19 years, and the most frequently encountered symptoms were represented by headaches, raised intracranial pressure syndrome, and motor deficits. More than 80% of the patients had a Karnofsky Performance Status (KPS) between 80 and 100, and the most associated comorbidities were cardiovascular and type 2 diabetes mellitus. A total of 88.70% of the patients had a single brain metastasis, and the most common localizations were the posterior fossa/cerebellum and frontal lobe. Gross-total resection was possible in 79.03% of the cases, while complications were recorded in 8.06%. Better survival rates were registered in patients of younger ages, with higher KPS, single BM, and smaller tumoral volumes, treated by gross-total resection and by a multimodal approach.

Conclusions: Notwithstanding significant advancements in the field of breast cancer, the prognosis of patients with brain metastases remains poor. However, a multimodal approach can prolong survival rates and improve outcomes, while in patients with acute clinical manifestations, neurosurgery remains the only immediate option to reverse the symptoms.

## Introduction

Breast cancer represents the second most frequent cause of brain metastases, after lung carcinoma [1,2], with an increasing reported incidence that is currently approximately 5.1% [3]. Although up to 16% of patients with breast cancer will develop brain metastases, particular molecular subtypes like human epidermal growth factor receptor 2 (HER2) positive and triple-negative breast cancer (TNCB) will have a greater predisposition for this event [4,5]. Cancerous cells need to penetrate the blood-brain barrier to metastasize to the brain, and this can only be achieved by adapting to various processes and acquiring specific features. Thus, the primary speculation sustains that the cancerous cells experience an epithelial-to-mesenchymal transition to pass into the blood circulation [6]. Subsequently, these cells will extravasate and reverse the mesenchymal-to-epithelial transition. Finally, they implant into the brain, start multiplying, and begin the neovascularization process, creating a new barrier called the brain tumor barrier [6]. It has been demonstrated that specific biomolecular changes, like HER2 status, sustain these complex processes and can have a major impact on the course of the disease [7].

The most reliable neuroimaging technique for preoperative evaluation remains cerebral magnetic resonance imaging (MRI), which provides considerable pieces of information [8].

Currently, the treatment for patients with brain metastases from breast cancer (BMBC) includes similar options to other brain malignancies [9]. The role of neurosurgical resection is indisputable in patients with acute symptoms due to mass effect or hydrocephalus and is the only therapeutical option that can immediately reverse the clinical manifestation [8]. However, other strategies include stereotactic radiosurgery, whole-brain radiotherapy, immunotherapeutic and chemotherapeutic agents [9]. The multimodal therapeutical approach has shown better outcomes in comparison to single approaches [9], mainly since specific targeted agents have been studied and developed in the last decade [10].

The main objective of the current study is to emphasize the role of neurosurgery in patients with BMBC by evaluating the outcomes after surgical excision with or without a multimodal approach. Furthermore, the study gives a strictly neurosurgical point of view regarding this disease by comparing different therapeutical approaches surrounding the surgery.

## Materials and methods

Study design

The present work is a retrospective study from reviewing the medical records from our institutional databases of 62 patients treated for BMBC in the 3rd Neurosurgical Department of Clinical Emergency Hospital “Bagdasar-Arseni”, Bucharest, Romania, between January 2013 and December 2023. The Research Ethics Committee of Clinical Emergency Hospital “Bagdasar-Arseni” has approved this study (No. 53407/11.04.2024). All the included patients have consented to the retrospectively collected data from the hospital’s databases and physical files.

Inclusion and exclusion criteria

The inclusion criteria were represented by: 1) breast cancer as their only primary malignancy; 2) patients over 18 years old with histopathologically confirmed BMBC after neurosurgical intervention in our clinic. The exclusion criteria were represented by 1) incomplete clinical data, 2) patients with metastases other than the brain, and 3) patients initially treated in other departments who eventually came in with tumoral recurrences.

Data collection

To select the patients, we interrogated the hospital’s digital databases for the terms “brain metastasis”, “brain metastases”, “breast cancer”, and “breast neoplasm”. Subsequently, we evaluated each patient’s physical files and thoroughly appraised their data, reviewing elements such as demographics, histopathology, neuroimaging features, clinicopathological data, neurosurgical treatment, and outcomes.

The collected data were categorized based on the type of analysis, and each clinical history, neurological examination, and perioperative data was reviewed to obtain correlations and draw statistical conclusions. The identified data for review was represented by age at diagnosis of breast cancer, age of diagnosis of BMBC, geographic region of origin, clinical symptoms at admission, KPS scale at admission, associated comorbidities, number of cerebral metastases, localization of breast cancer, cerebral localization of BM, grade of tumoral resection, postoperative complications, and other treatments like gamma-knife radiosurgery, radiotherapy, and chemotherapy. This data allowed us to further assess survival rates, which were compared based on the recorded results.

To ensure data accuracy, both the physical files and the hospital’s databases were thoroughly assessed, while records with incomplete or ambiguous information were excluded. All collected data were anonymized to uphold patient confidentiality and ensure compliance with ethical research standards.

Statistical analysis

Correlations between the acquired data were performed to draw statistical conclusions. All data analyses were performed using GraphPad Prism 10.2.1 (339) version software and Microsoft Excel version 16.89.1. The Chi-square test (Fisher Exact test) was applied to assess the variables' differences and correlate normally distributed data. At the same time, a p-value of less than 0.05 was considered statistically significant. The overall survival was calculated from the date of histopathological diagnosis to the date of death, and the method of choice to estimate the survival rate was the Kaplan-Meier method (Log-rank Mantel-Cox test, Gehan-Breslow-Wilcoxon test, and Mantel-Haenszel were used to compare the survival curves).

## Results

Demographic Profile, Clinicopathological Features, and Correlation Analysis

The total number of selected patients who accomplished the inclusion requirements for the study was 62 (N=62), all women. The median age at diagnosis of BMBC was 57 years, ranging from a minimum of 35 years old to a maximum of 80 years old. Some of the patients were previously diagnosed with breast cancer and received treatment. In contrast, others didn’t have a diagnosis of malignancy before being admitted to the hospital, but this was established after the neurosurgical intervention. The median age at diagnosis of breast cancer was 54.5 years, ranging from a minimum of 30 years to a maximum of 78 years. The characteristics of the patients in the study group are summarized in Table 1.

**Table 1:** Patients’ characteristics in the study group BM: brain metastases; BMBC: brain metastases from breast cancer

Characteristics	Number of patients (N)	Percentage (%)
Age at diagnosis of BMBC		
Median (years)/Mean 𝜇 (years) – 57/57.19		
Minimum/Maximum (years) (+/-SD) – 35/80 (+/-11.76)		
Age at diagnosis of breast cancer		
Median (years)/Mean 𝜇 (years) – 54.5/54.51		
Minimum/Maximum (years) (+/-SD) – 30/78 (+/-12.27)		
Geographic Area		
Urban	46	74.19%
Rural	16	25.80%
Clinical Symptoms at Admission		
Headache	46	74.19%
Raised intracranial pressure syndrome (RIPS)	20	32.25%
Motor deficits	12	19.35%
Seizures	9	14.51%
Altered mental status	8	12.90%
Vertigo	7	11.29%
Visual disturbances	4	6.45%
Nystagmus	4	6.45%
Hydrocephalus	3	4.83%
Aphasia/Dysphasia	2	3.22%
Dysarthria	2	3.22%
Dysphagia	2	3.22%
Facial nerve paralysis	2	3.22%
Gait disturbances	2	3.22%
Frontal lobe syndrome	1	1.61%
Amnesia	1	1.61%
Trigeminal neuralgia	1	1.61%
Hypoacusis	1	1.61%
Dysmetria	1	1.61%
Ataxia	1	1.61%
Hypothalamic dysfunction	1	1.61%
Karnofsky Performance Status (KPS) Scale at Admission		
<40	3	4.83%
50-70	9	14.51%
80-100	50	80.64%
Comorbidities		
Cardiovascular	13	20.96%
Diabetes mellitus type 2	6	9.67%
Obesity	3	4.83%
Other	2	3.22%
Number of Cerebral Metastases		
Single BM	55	88.70%
More than one BM	7	11.29%
Localization of Breast Cancer		
Unilateral	59	95.16%
Bilateral	3	4.83%
Cerebral Localization of BM		
Posterior fossa/Cerebellum	18	29.03%
Frontal lobe	12	19.35%
Frontoparietal	5	8.06%
Frontotemporal	2	3.22%
Temporal lobe	5	8.06%
Temporoparietal	3	4.83%
Temporo-occipital	1	1.61%
Parietal lobe	3	4.83%
Parieto-occipital	3	4.83%
Occipital lobe	3	4.83%
Multiple localization	7	11.29%
Tumoral volumes		
Median (cm^3^)/Mean 𝜇 (cm^3^) 37.3/53.5		
Minimum (cm^3^)/Maximum (cm^3^) (+/-SD) 12.6/199.3 (+/-40.4)		
Grade of Tumoral Resection		
Gross-total resection	49	79.03%
Subtotal resection	9	14.51%
Biopsy	4	6.45%
Postoperative Complications		
Yes	5	8.06%
No	57	91.93%
Gamma-Knife Radiosurgery		
Yes	10	16.12%
No	52	83.87%
Radiotherapy		
Yes	26	41.93%
No/Unknown	36	58.06%
Chemotherapy		
Yes	34	54.83%
No/Unknown	28	45.16%

When split into age groups, the results showed a predominance of patients in their 5th decade. No patients were reported in the 20-29-year-old group, 3 patients (4.83%) were in the 30-39-year-old group, 15 patients (24.19%) were in the 40-49-year-old group, 18 patients (29.03%) were in the 50-59-year-old group, 16 patients (25.80%) were recorded in the 60-69-year-old group, 9 patients (14.51%) were in the 70-79-year-old group and 1 patient (1.61%) was 80 years old. Regarding the tumoral volumes in the study group, the median volume was 37.3 cm^3^, ranging from 12.6 cm^3^ to 199.3 cm^3^.

**Figure 1:**
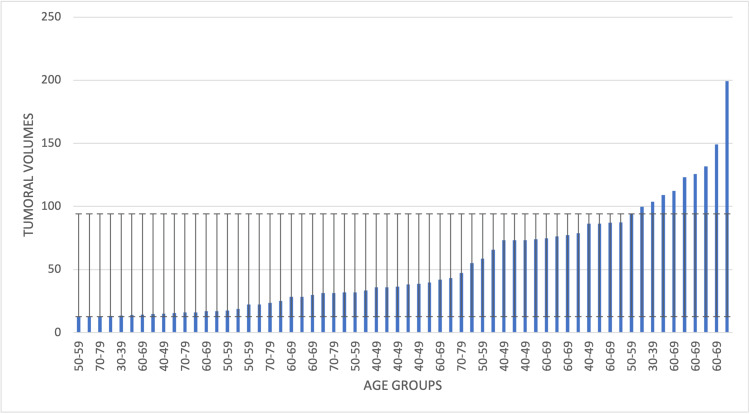
Tumoral volumes of BMBC distributed by age groups The image highlights the presence of the largest tumoral volumes in patients from the 50-59 and 60-69 age groups, as well as the smallest tumoral volumes in patients over 80 years old and from the 30-39 years old group. BMBC: brain metastases from breast cancer The image was created by the author of this article.

Patients in the 50-59 age group had the largest lesional dimensions, followed by patients in the 60-69 and 40-49 age groups, while patients in the 30-39 and 80+ age groups had the smallest tumoral volumes in the study group (Figure 1).

**Figure 2:**
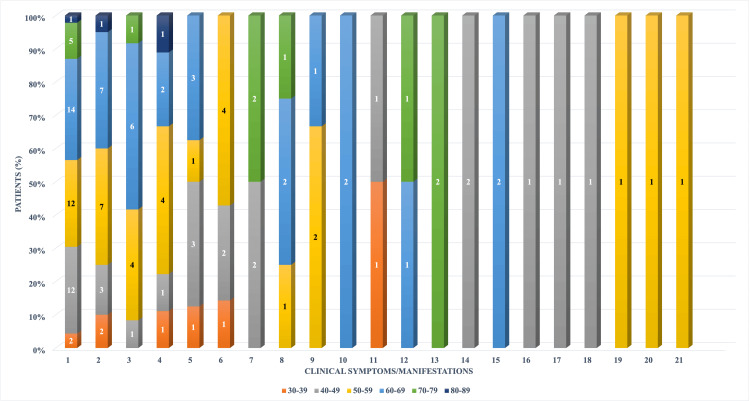
The distribution of symptoms in the study group concerning age groups 1 – Headache; 2 – RIPS; 3 – Motor deficits; 4 – Seizures; 5 – Altered mental status; 6 – Vertigo; 7 – Visual disturbances; 8 – Nystagmus; 9 – Hydrocephalus; 10 – Aphasia/Dysphasia; 11 – Dysarthria; 12 – Dysphagia; 13 – Facial nerve paralysis; 14 – Gait disturbances; 15 – Frontal lobe syndrome; 16 – Amnesia; 17 – Trigeminal neuralgia; 18 – Hypoacusis; 19 – Dysmetria; 20 – Ataxia; 21 – Hypothalamic dysfunction RIPS: raised intracranial pressure syndrome The image was created by the author of this article.

The symptomatology in BMBC depends on tumoral volume and localization in the brain. The most frequent symptoms recorded in the study group were represented by headaches (35%), followed by raised intracranial pressure syndrome (RIPS) (15%), motor deficits (9%), seizures (7%), altered mental status (6%), and vertigo (6%). Other symptoms were less frequent and have already been specified in Table 2. When distributed by age groups and symptoms, patients in the 60-69 and 50-59 age groups had more severe and multiple symptoms. Patients in the 30-39 and 70-79 age groups had less severe and singular symptoms (Figure 2).

Neurosurgical results

In the study group, neurosurgical intervention was indicated when there was a single BM or a small number of lesions. Given the pivotal role of neurological improvement and local decompression, which can be lifesaving, neurosurgical intervention was the main therapeutic option in symptomatic patients, especially in acute onsets. Sometimes, when multiple BMs were discovered, only the symptomatic lesion was relevant for neurosurgery, while others were treated by radiosurgery. It is worth mentioning that the excision grade depended mainly on tumoral localization. Although advanced neurosurgical techniques are available for a safe maximal resection, gross-total resection is not always feasible. Thus, for BMs located in eloquent or deep areas of the brain, subtotal resection or biopsy was the only neurosurgical option. Furthermore, the neurosurgical intervention was supported by medical treatment for seizures, cerebral edema, and pain.

Regarding the neurosurgical results, gross-total resection (GTR) was performed in 49 (79.03%) patients, while subtotal resection (STR) or biopsy was performed in 13 (20.96%) patients.

Complications related to neurosurgical intervention in the study group have been recorded in five cases, represented by hemorrhage in two (3.22%) cases, hydrocephalus in two (3.22%) cases, and wound-related complications in one (1.61%) case, while no surgical-related mortality was recorded. When correlating the resection grade with postoperative complications, it was found that patients with GTR were more likely to develop complications; p=0.3340, t= 1.667, df=1.

The main histological subtypes were invasive ductal carcinoma (IDC) in 53 (85.4%) patients, followed by invasive lobular carcinoma (ILC) in seven (11.2%) patients, while two (3.2%) patients had other histological subtypes. An insufficient number of patients had a molecular subtype assessment, so this feature was not included in the statistical analysis.

Survival analysis

**Figure 3:**
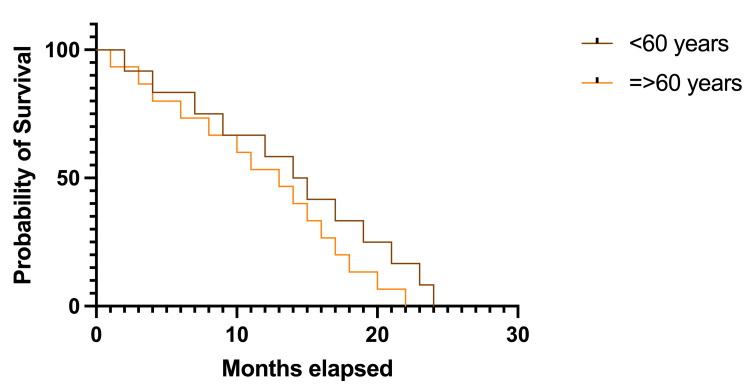
Kaplan-Meier plot describing the survival rate according to age groups The image was created by the author of this article.

The survival analysis in the study group was based on different prognostic factors like age, Karnofsky Performance Status (KPS) score at admission, tumoral dimensions, grade of resections, number of lesions, and therapeutic approach. Longer median survival rates were observed, analyzing patients younger than 60 years compared with patients 60 years or older. Patients younger than 60 had a median survival rate of 14.5 months (Hazard ratio (HR) 1.115, 95% CI 0.522-2.383), while older patients had a median survival rate of 13 months (HR 0.896, 95% CI 0.419-1.915). However, the correlation was not statistically significant, p=0.2391 (Figure 3).

**Figure 4:**
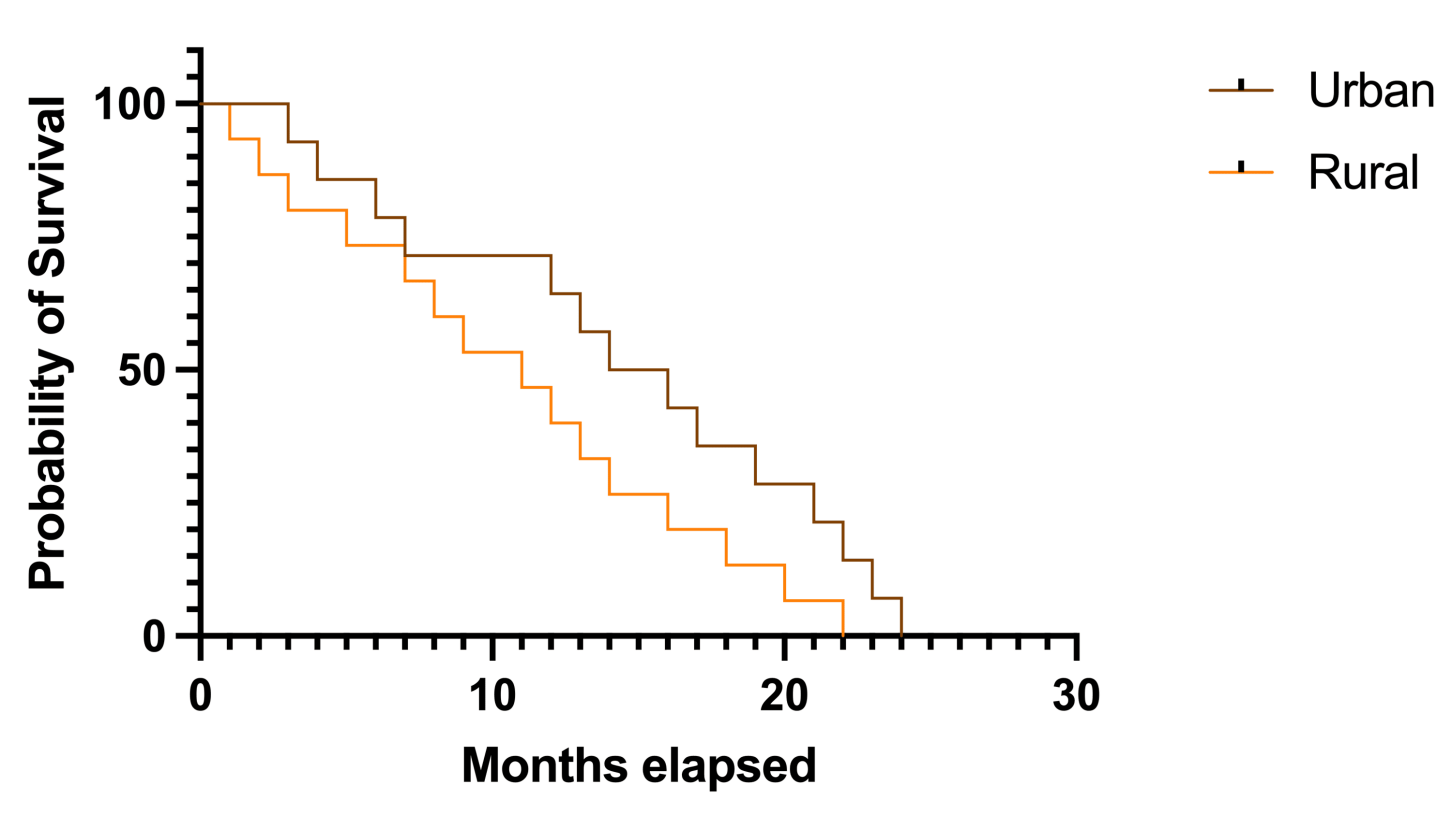
Kaplan-Meier plot describing survival rates according to geographical localization The image was created by the author of this article.

There was a non-significant trend toward longer survival rates in patients from urban settings: the median survival rate was 15.0 months for urban settings, HR 1.364, 95% CI 0.658-2.825 versus 11.0 months for rural areas, HR 0.733, 95% CI 0.354-1.519; p=0.102 (Figure 4).

**Figure 5:**
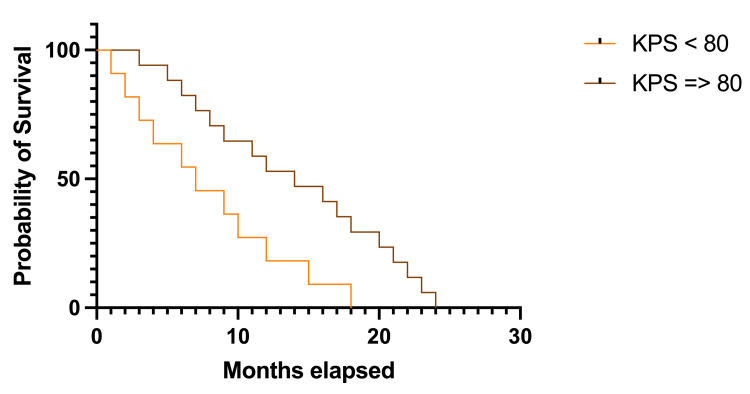
Kaplan-Meier plot describing survival rates based on KPS at admission KPS: Karnofsky Performance Status The image was created by the author of this article.

Two groups were created to analyze patients’ survival rates based on KPS admission scores (Figure 5). The first group was represented by patients with a KPS score greater than 80 (50 patients, representing 80.64%), and the second group included patients with a KPS score equal to or lower than 80 (12 patients, representing 19.35%). Statistically significant results were recorded, while survival rates were lowered by half in patients from the first group (7.0 months, HR 0.302, 95% CI 0.115-0.792 versus 14.0 months, HR 3.301, 95% CI 1.262-8.639); p=0.015, df=1.

**Figure 6:**
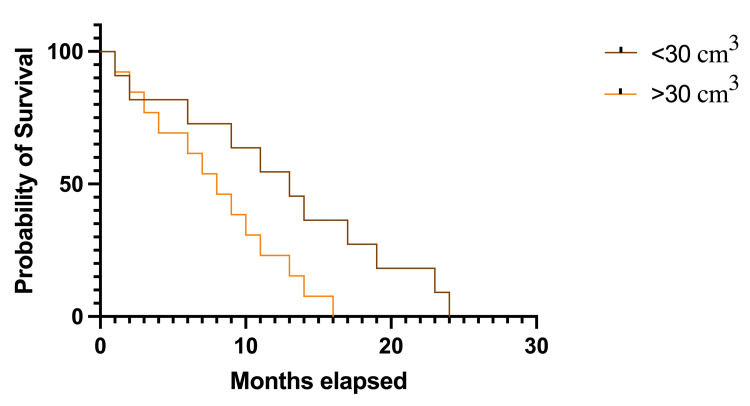
Kaplan-Meier plot describing survival rates in patients with different tumoral volumes The image was created by the author of this article.

Tumoral dimensions were also included in the survival analysis after being categorized as smaller than 30 cm3 and equal to or larger than 30 cm3. Patients with greater tumoral dimensions had a median survival rate of 8.0 months (HR 0.615, 95% CI 0.275-1.374) compared to patients with smaller tumors, which had a median survival rate of 13.0 months (HR 1.625, 95% CI 0.728-3.627), and this was statistically significant: p=0.038, df=1 (Figure 6).

**Figure 7:**
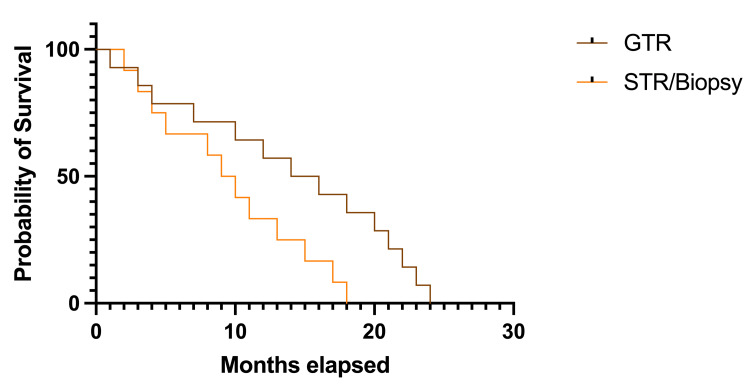
Kaplan-Meier plot describing survival rates in patients with different tumoral resection grades GTR: gross-total resection; STR: subtotal resection The image was created by the author of this article.

There was a significant trend toward longer survival rates in patients with GTR, where the median survival rate was 15.0 months (HR 1.579, 95% CI 0.730-3.414), versus 9.5 months in other patients (HR 0.633, 95%CI 0.292-1.369); p=0.036, df=1 (Figure 7).

**Figure 8:**
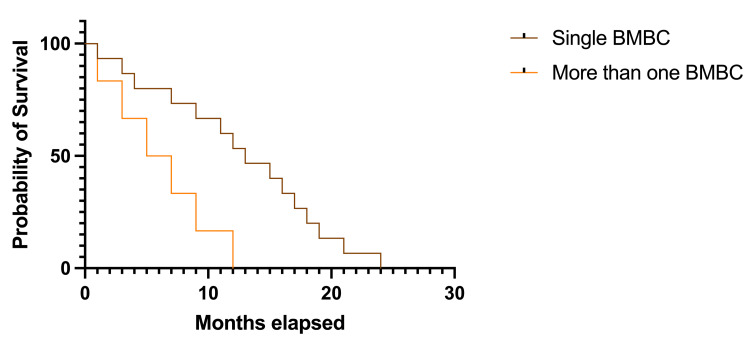
Kaplan-Meier plot describing survival rates in patients with single or multiple cerebral BMBC BMBC: brain metastases from breast cancer The image was created by the author of this article.

It is observed a significant trend toward longer survival in patients with a single lesion (55 patients - 88.70% - a median survival rate of 13.0 months, HR 2.167, 95% CI 0.840-5.584) versus patients with more than one lesion (7 patients - 11.29% - 6.0 months, HR 0.461, 95% CI 0.179-1.190); p=0.0277, df=1 (Figure 8).

**Figure 9:**
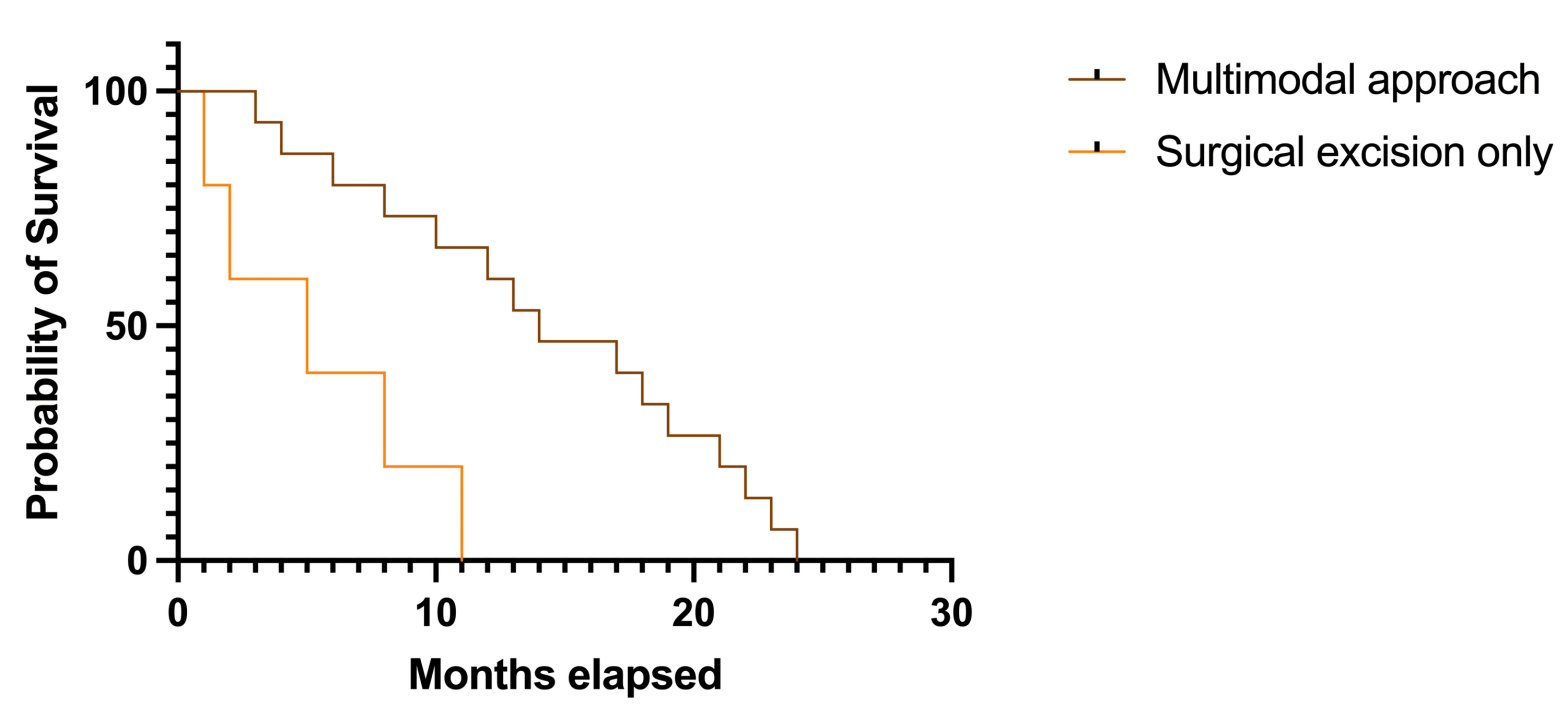
Kaplan-Meier plot describing survival rates based on different therapeutic approaches in the study group The image was created by the author of this article.

Considering the therapeutic approach in the study group, survival rates were longer in patients treated by a multimodal approach (neurosurgical excision, radiotherapy, chemotherapy) than in patients treated only by neurosurgical excision (Figure 9). The trend toward longer survival rates was statistically significant (14.0 months, HR 3.874, 95% CI 0.076-19.630 versus 5.0 months, HR 0.258, 95% CI 0.050-1.308); p=0.026, df=1.

## Discussion

Although breast cancer is the second most frequent after lung cancer, BMBC represents a step reserved usually for advanced cases, which account for approximately 5.1% [10]. Furthermore, various risk factors have been described to contribute to the metastatic process in the brain, like the patient’s age, molecular and histological characteristics, or breast tumor dimensions [11]. Other factors may impact the course of this disease, such as the KPS score at admission, the number of brain metastases, the grade of tumoral resection, and the therapeutic approach.

Sixty-two patients with newly diagnosed BMBC were treated in the department and selected to be included in this study. All the patients were females, and the mean age was 57.19 years (median age 57 years, min. 35, max. 80, SD±11.76), and these results are consistent with previous and current scientific literature [12-14]. In addition, the study found that patients in the 60-69 and 50-59 age groups had more severe and multiple symptoms, while patients in the 30-39 and 70-79 age groups had less severe and singular symptoms. The most frequent symptoms at admission in the clinic were represented by headaches (74.19%), followed by raised intracranial pressure syndrome (32.25%), motor deficits (19.35%), and seizures (14.51%). Similar results were reported by Rostami et al. in a comprehensive review of the medical literature, where 14,599 patients were included, and headache, hemiparesis, and seizures were among the most frequently reported symptoms [15].

Tumor volumes of BMBC were often considered important prognostic factors, and the study is consistent with these proven theories. The study population’s median tumoral volume was 37.3 cm^3^, ranging from 12.6 cm^3^ to 199.3 cm^3^ (SD±40.4). Furthermore, when distributed by age groups, patients in the 50-59 and 60-69 age groups had the largest lesional volumes, while patients in the 30-39 and 80+ age groups had the smallest tumoral volumes. This is especially important considering that the lesional volume can influence survival rates. In the present analysis, the tumoral volumes are split into two categories: smaller than 30 cm^3^ and equal to or larger than 30 cm^3^. The results showed a significantly longer median survival rate in patients with smaller tumors (13.0 months) than others (8.0 months). The results are similar to those of other studies on this matter. In a recent study from 2024, Yakar and Etiz stated that larger BMBCs are more prone to vasogenic edema. Thus, they have a higher risk of neurologic aggravation [16]. Dohm et al. stated that larger brain lesions are included in the higher-risk group, and the likelihood of death due to neurological causes is 20% in BMBC [17]. In this manner, Hackshaw et al. concluded that larger tumoral volumes are a prognostic factor for shorter survival rates [18].

Limon et al. evaluated 59 consecutive patients with BMBC. They concluded that smaller tumoral volumes, higher doses of stereotactic radiosurgery (SRS), and lower brain volumes receiving more than 12 Gy were associated with longer survival rates in individuals with multiple tumoral lesions. Furthermore, a mean dose of more than 19 Gy to the entire planning total volume and a total planning volume of less than 10 ccs in patients treated with single fraction SRS was also correlated with longer survival rates [19].

Regarding the geographic area of origin, in the study population, 74.19% of the cases were from urban settings, and 25.80% were from rural areas. While this might not be of great interest in other circumstances, it is worth mentioning that in Romania, the greatest neurosurgical and oncological centers are in urban settings, while in rural areas, it might sometimes be challenging to find a medical facility that can provide optimal management. Young patients have better opportunities to access medical aid, while older patients need additional help. Although the analysis was not statistically significant (p=0.102), it was observed that patients from urban settings had longer survival rates when compared to others (median survival rate of 15.0 months versus median survival rate of 11.0 months).

The KPS score at admission in the department was less than 40 in 4.83% of the cases, between 50 and 70 in 14.51%, and between 80 and 100 in 80.64% of cases. Thus, 50 (80.64%) patients had a KPS greater than 80, while 12 (19.35%) patients had a KPS equal to or lower than 80. The analysis concluded longer survival rates in patients with better KPS scores, and these results were statistically significant (14.0 months versus 7.0 months, p=0.015). Furthermore, the results were consistent with prior studies. In a study from 2022, Freeman et al. stated that better KPS scores were a strong predictor for long survival in patients with BMBC [20]. Similarly, Michel et al. concluded that longer intervals between BC diagnosis and BMBC development, a specific molecular subtype, and KPS equal to or greater than 90 were predictive factors for long-term survival [21]. A few factors should be considered regarding the threshold of dichotomization of KPS. The KPS was developed to evaluate the health-related quality of life in cancer patients and the ability to independently perform daily activities and track the impact of cancer on these patients [22]. A score of 0 is assigned if the patient can’t perform any daily activity, while a maximum score of 100 is assigned if the patient can independently perform every daily activity measured by the score [23]. However, an assigned score of 80 points is the last one to describe the ability to carry out activities, even light or sedentary, while an assigned score of 70 points is the first one to describe the inability to perform any work activity, even if the ability for self-care is preserved [24]. Thus, the threshold for dichotomization in the present study is based on this reasoning, as it has already been done in previous similar studies [25].

In a recent study from 2024, Mashiach et al. evaluated 190 patients with BMBC and concluded that a KPS of less than 80 at first SRS is a predicting factor of death [26].

Many studies regarding BMBC have used the number of brain metastases as a prognostic factor. In the study group, 88.70% of the patients had a single BMBC, while only 11.29% had more than one. Longer survival rates were observed in the first cases compared to others (13.0 months versus 6.0 months), and the difference was statistically significant (p=0.0277).

Hackshaw et al. concluded that along with other factors, less than three cerebral lesions predict a longer survival [18]. The same conclusion was cited even earlier by Eichler et al. who stated that three or fewer brain metastases, no systemic disease, and local control are good predictors of survival rates [27]. In a study from 2023 regarding the assessment of survival rates in patients with BMBC, Riecke et al. demonstrated that a lower number of cerebral lesions is associated with long-term survival in these patients [13]. Simsek et al. observed that multiple BMBC and leptomeningeal involvement were associated with shorter survival rates, and they suggested a possible reason for the lack of curative therapeutical options for these cases [28].

Even though BMBC has intricate therapeutic management, neurosurgical intervention is the cornerstone to prompt disease control and symptom alleviation [29]. In the study group, 12.90% of patients had an altered mental status. In comparison, more than 50% had at least one neurological symptom that required immediate neurosurgical intervention, or other therapeutical options would not cure that.

Regarding the grade of tumoral resection, the purpose was a safe maximal excision to minimize relapses, and the main goal was to reduce the intracranial mass effect in patients with acute onset of neurological symptoms. Although GTR was possible in 79.03% of cases due to several factors (like anatomical localization or infiltrative feature), a subtotal resection was performed in the rest of the patients from the study group. When analyzing survival rates according to the grade of tumoral excision, a significant trend was observed toward longer survival in patients with maximal resections compared to others (15.0 months versus 9.5 months).

However, the neurosurgical approach is not risk-free, and postoperative complications might appear. In this study, it was recorded a total of five cases of postoperative complications, which were represented by hemorrhage in two (3.22%) cases, hydrocephalus in two (3.22%) cases, and wound-related complications in one (1.61%) case. When analyzing postoperative complications correlated to the resection grade, it was observed that patients with GTR were more likely to develop complications. In contrast, patients with subtotal resection or biopsy had fewer chances, although the association was not statistically significant.

A histopathological result was obtained in all the patients in the postoperative settings. The main histological subtypes recorded were represented by IDC in 85.4% of cases, ILC in 11.2%, and other histological subtypes in 3.2%. When correlated with postoperative complications, it was observed that patients with IDC were more likely to have complications than others, but the correlation was not statistically significant. The main reason for the insignificant correlation is the significant numerical difference between the three groups (IDC, ILC, and other subtypes).

A recent study from 2024, written by Mubarak et al., analyzed the 5-year survival impact of the histologic subtype of breast cancer and found no difference in the survival rates for these patients [30].

Some authors may doubt the efficacy of chemotherapeutic agents [31]. Saatian et al. concluded that some chemotherapeutic agents, such as paclitaxel and lapatinib, may increase the permeability of the blood-brain barrier and blood-cerebrospinal fluid barrier, not only allowing tumoral cells to metastasize but also influencing the development of an Alzheimer’s-like pathology through higher release of Tau [32]. However, compared to standard chemotherapy, new targeted therapies have been proven to have fewer adverse events and shorter administration intervals while providing better results, especially when combined with other therapies [33].

It has been stated that some agents might easily penetrate the brain, yet they have a decreased activity when it comes to BM [6]. This study did not separate chemotherapy and radiotherapy as therapeutic options. Still, it included them in the multimodal approach along with neurosurgical intervention while comparing this type of management with neurosurgery alone. The results showed longer survival rates in patients treated by a multimodal approach by more than half, and the results were statistically significant. Patients treated only by neurosurgical excision had a median survival rate of 5.0 months, while for the rest of the cases, the recorded median survival was 14.0 months.

The general opinions in medical literature might be contrasting regarding the multimodal approach. Bailleux et al. stated that in patients with post-radiotherapy progressive cerebral metastases, the results are worse if systemic therapy is involved [6]. Niwińska et al. concluded a statistically significant difference when comparing survival rates in patients treated with and without systemic treatment after radiotherapy. Thus, patients with systemic therapy and radiotherapy had a median survival rate of 10 months, while patients treated only with radiotherapy had a median survival rate of 3 months [34].

The benefits of a multimodal approach have also been supported by Simonsen et al., who further suggests the individualization of therapeutical management based on described prognostic factors [35].

Although there are no specific criteria regarding predictors of survival after SRS in BMBC patients, Wilson et al. published a retrospective analysis of 91 consecutive patients with BMBC. The authors concluded that worse prognosis was associated with factors like largest tumoral volumes (>10 cm^3^) or progressive extracranial disease, while stable extracranial disease was a factor associated with longer survival rates [36].

A major indicator of the terminal stage of BMBC is represented by unmanageable neurological manifestations even after a multimodal approach, and in such cases, palliative care remains the last option [37].

Notwithstanding, the prognosis of BMBC can be highly influenced by the molecular subtype of breast cancer [38,39]. Very few patients had a molecular assessment, which did not allow statistical analysis. Thus, this is the main limitation of the study. Another limitation might be the exclusion of some patients with BMBC if they had other metastatic sites besides the brain. Including only the cases with exclusively brain metastases, the neurosurgical results were evaluated more accurately, without any major biases, as many patients can worsen their clinical course, and, in some cases, it would be difficult to differentiate if the aggravation was due to the brain metastases or the other metastases.

## Conclusions

Brain metastases in patients with breast cancer are an indicator of an advanced disease. In acute neurological symptoms or even aggravation, neurosurgical intervention might be the only therapeutical solution initially in the short term. Survival rates can be correlated with the patient’s age, number, and size of BMBC, grade of tumoral excision, and therapeutical management. Although various strategies have been studied, discovered, and applied to improve the clinical course of BMBC, and some limited long-term survival cases have been reported, the prognosis remains mainly poor. Longer survival rates were recorded in patients of younger age, with KPS scores greater than 80 points, with smaller tumoral volumes, with GTR, and a single BM, that were treated by a multimodal approach. Notwithstanding the numerous studies and advancements from the last decades regarding patients with BMBC, further research is needed to manage the disease better and improve the current dismal prognosis.
